# Supplementary material for: Experimental Epileptogenesis in a Cell Culture Model of Primary Neurons from Rat Brain: A Temporal Multi-Scale Study
Source: Cells. 2021 Nov 3;10(11):3004. doi: 10.3390/cells10113004 (PMC8616120; doi:10.3390/cells10113004)
Supplement: Supplementary file 1 [file cells-10-03004-s001.zip › cells-1396782-supplementary.pdf]

## Supplementary Material

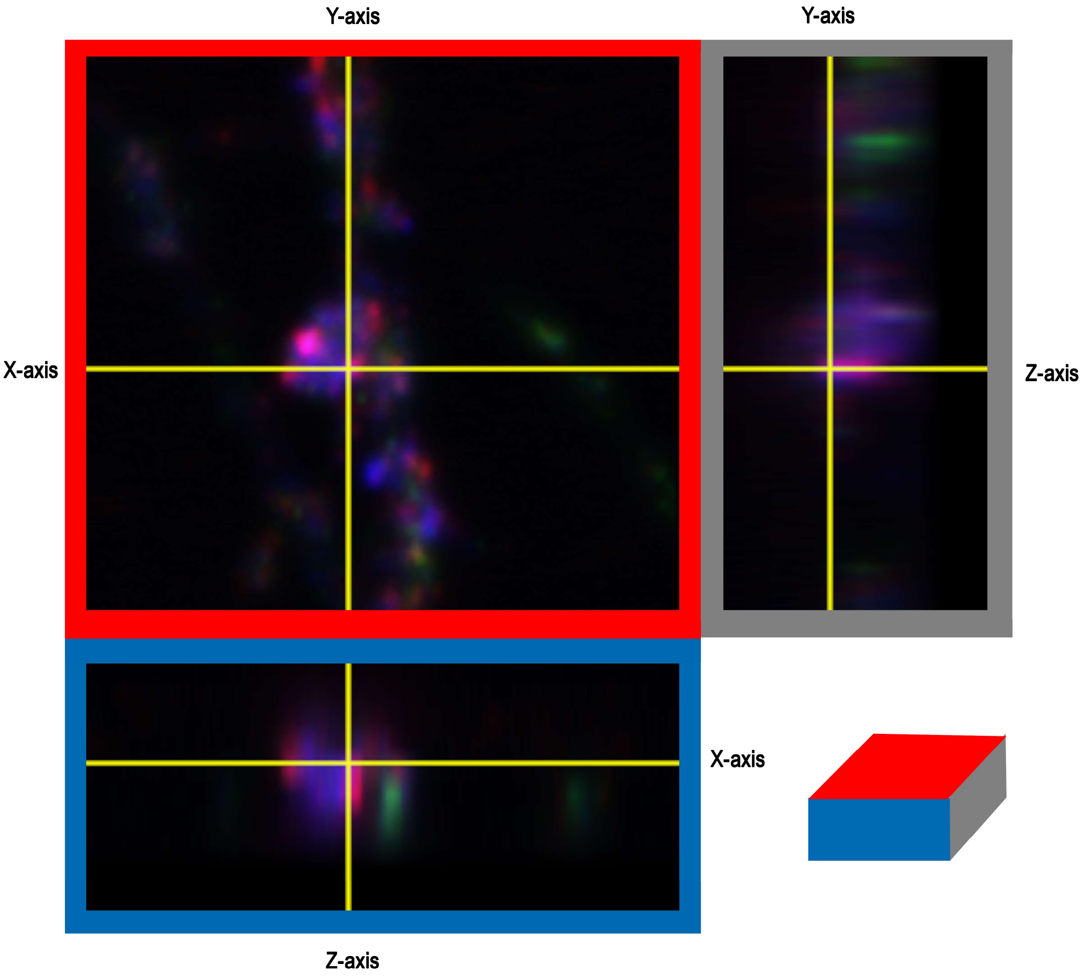


**Figure S1.** Z-stack of a confocal image showing co-localization of Vglut1 (blue) and Vgat (green) with Bassoon (red) marking active synaptic terminals.
